# Formalin Treatment of Malignant Tumors

**Published:** 1915-11

**Authors:** 


**Formalin Treatment of Malignant Tumors.** R. Lataste, These de Bordeaux, 1913, quoted in Arch. d'Elect. Med., Aug. 1915, tried this method, favorably described by Laurent of Brussels in 6 cases. While agreeing that formalin does produce coagulation, he finds the action purely local, the tumors extending at points not directly reached by the injections.

